# The use of antibiotic-loaded calcium sulphate beads in debridement, antibiotics, and implant retention (DAIR) for periprosthetic infections: a retrospective comparative cohort on outcome

**DOI:** 10.2340/17453674.2024.42360

**Published:** 2024-11-29

**Authors:** Irene Katharina SIGMUND, Antony J R PALMER, Andrew J HOTCHEN, Martin A MCNALLY, Bernadette C YOUNG, Abtin ALVAND, Adrian TAYLOR, Benjamin John Lee KENDRICK

**Affiliations:** 1 Nuffield Orthopaedic Centre, Oxford University Hospitals, Oxford, UK; 2 Department of Orthopaedics, Medical University of Vienna, Vienna, Austria; 3 Nuffield Department of Orthopaedics, Rheumatology, and Musculoskeletal Sciences, University of Oxford, Oxford, UK; 4 Nuffield Department of Medicine, University of Oxford, Oxford, UK

## Abstract

**Background and purpose:**

We aimed to compare the effect of calcium sulphate (CS) beads loaded with antibiotics on infection eradication in debridement, antibiotics, and implant retention (DAIR) of periprosthetic joint infection relative to DAIR without local antibiotics delivery.

**Methods:**

176 patients with hip or knee arthroplasty undergoing DAIR were retrospectively identified and divided into a bead group (n = 102) and a control group (n = 74). Infections were classified as early postoperative, acute hematogenous, and chronic. Logistic regression analyses were performed on the use of CS beads. Revision-free and infection-free survival was estimated using Kaplan–Meier analysis.

**Results:**

Reinfection occurred in 36% of the bead group, and 39% of the control group (odds ratio [OR] 0.9, 95% confidence interval [CI] 0.5– 1.6); reoperation rates were 34% and 43% (OR 0.7, CI 0.4–1.3). Kaplan–Meier analysis showed no statistically significant difference between the 2 groups regarding infection-free (HR 1.1, CI 0.7–1.8) and revision-free (HR 1.1, CI 0.7–1.9) survival rates. In acute hematogenous PJIs, reinfection (29% vs 56%, OR 0.3, CI 0.1–1.1) and reoperation rates (25% vs 61%, OR 0.2, CI 0.1–0.8) were reduced when CS beads were used; Kaplan–Meier analysis revealed higher infection-free (HR 0.5, CI 0.2–1.4) and revision-free (HR 0.5, CI 0.2–1.3) survival rates in the bead group but not of statistical significance. Wound drainage was not increased by CS beads use (OR 1.0, CI 0.99–1.01), but hypercalcemia was seen in 9% in the bead group.

**Conclusion:**

DAIR with antibiotic-loaded CS beads did not improve outcome in early postoperative and chronic PJIs, but reduced the reoperation rate in acute haematogenous infections with similar results compared with early postoperative PJIs.

Biodegradable calcium sulphate (CS) beads loaded with antibiotics are used for periprosthetic joint infection (PJI) treatment. They may enhance success by local bioburden and biofilm reduction. Due to their complete absorption over a period of several weeks, they provide a constant and complete release of their antimicrobial content locally without the need for further surgery [1,2]. In-vitro studies have demonstrated good elution characteristics of CS beads, leading to a high antimicrobial concentration surpassing the minimal inhibitory concentration for common PJI pathogens and superior biofilm reduction/eradication [3-7]. Due to these beneficial properties, they may also reduce the intraarticular microbial load and biofilm formation on component surfaces and tissues. Therefore, the local use of CS as delivery agent for antibiotics has received strong interest in the orthopedic community to improve debridement, antibiotics, and implant retention (DAIR) outcomes [8-15]. Although some authors have reported high success rates (85–88%) in patients undergoing DAIR combined with application of antibiotic-loaded absorbable CS beads [11,13,14], others have shown inferior results (52–55%) [8,12]. Additionally, specific complications were reported to be associated with CS use including hypercalcemia, prolonged wound drainage, and heterotopic ossification [1,16,17]. The number of patients treated with DAIR in each of these studies was small. Due to conflicting results and paucity of published evidence, the efficacy of DAIR procedures with CS beads remains unclear.

Therefore, we aimed to assess the outcome of DAIR with or without the use of antibiotic-loaded absorbable CS beads for hip and knee PJI treatment. Outcomes included reinfection, reoperation, wound drainage, hypercalcemia, and heterotopic ossification.

## Methods

### Study design and population

This retrospective comparative cohort study was performed at a tertiary orthopedic hospital that specializes in PJI treatment, and is reported according to STROBE guidelines. Consecutive patients undergoing DAIR for periprosthetic hip or knee infections (defined by the European Bone and Joint Infection Society [EBJIS] definition of PJI [18]) between 2010 and 2020 were identified and classified into a bead group (DAIR with locally applied antibiotic loaded CS beads, 2015–2020) or control group (DAIR without local antibiotics, 2010–2014). PJIs were classified as early postoperative (< 4 weeks since surgery), acute hematogenous (< 3 weeks of symptoms after an uneventful postoperative period), or chronic (symptoms that persist for > 3 weeks and are beyond the early postoperative period) infections [19]. Patients with prostheses due to an oncological condition, patients with a washout due to another reason than infection, patients with missing data, patients with a unicompartmental knee prosthesis or patella-femoral resurfacing, patients with a Girdlestone hip, and patients with follow-up shorter than 12 months (without reaching an endpoint of reinfection or reoperation) were excluded. Cases were defined as uncomplicated or complex (including limited options) based on the Joint-Specific, Bone involvement, Anti-microbial options, Coverage of the soft tissues, Host status (JS-BACH) classification [20].

### Surgical procedure

**Figure 1:**
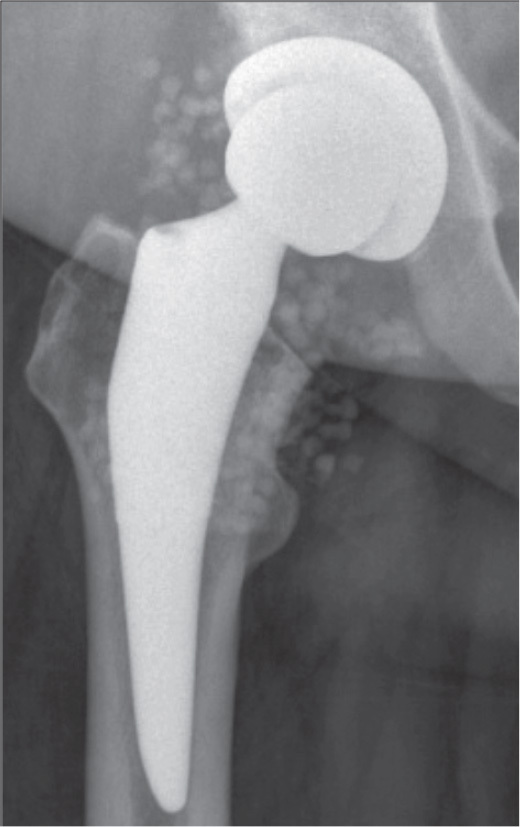
Anteroposterior radiograph after a DAIR procedure with application of calcium sulphate beads CS of 20 mL antibiotic-loaded rapid cure powder mixed with 2 g vancomycin and 480 mg gentamicin for an early postoperative periprosthetic hip infection with *Enterobacter cloacae* in a 57-year-old female patient.

**Figure 2:**
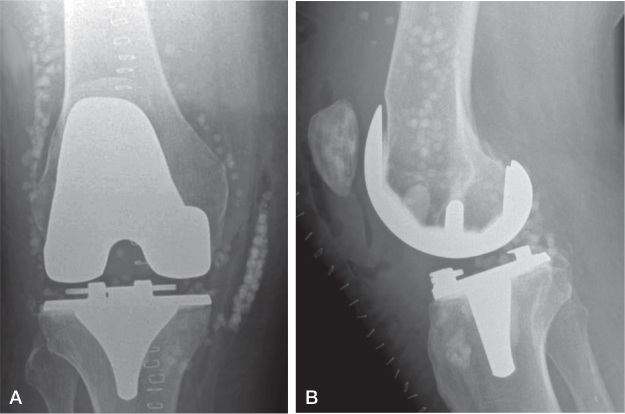
(A) Anteroposterior and (B) lateral radiograph after a DAIR procedure with application of calcium sulphate beads of 10 mL antibiotic-loaded rapid cure powder mixed with 1 g vancomycin and 240 mg gentamicin for an acute hematogenous periprosthetic knee infection with *Streptococcus dysgalactiae* in a 68-year-old male patient.

The DAIR technique at our institution has been described in detail in the past [21], but a summary of the important steps is given below. All procedures had standardized tissue sampling (5 samples for microbiology, 5 samples for histology). For each sample, new sterile instruments were used to avoid cross-contamination. Afterwards, a thorough debridement including synovectomy and excision of macroscopically infected and necrotic tissue was performed. The prosthetic–bone interface was carefully inspected, and the prosthesis tested for stability. Only in stable and soundly fixed implants was a DAIR procedure performed. The joint, surfaces of the retained implants, and soft tissues were washed with either 0.05% chlorhexidine or aqueous betadine based on surgeon preference. Surgeon discretion and availability of new mobile components determined whether an exchange of mobile parts took place (Table 1). In the bead group, the CS paste (Stimulan Rapid Cure, Biocomposites, Keele, UK) was prepared according to the manufacturer’s instructions by mixing 10 mL CS rapid cure powder with 1 g vancomycin and 240 mg gentamicin. After thorough irrigation of the joint, the cured CS beads were placed into the joint (hip: inferior to the acetabulum and around the proximal femur, knee: medial and lateral gutter and posterior capsule, Figures 1 and 2). CS beads of 10 mL antibiotic-loaded rapid cure powder were applied in 53 (52%), 20 mL in 43 (42%), 30 mL in 4 (4%), and 40 mL in 2 (2%) cases. In 3 patients, additional antibiotics were added according to the antibiogram and infectious diseases physician’s recommendation (patient 1: 10 mL + 1 g vancomycin, 10 mL + 200 mg amphotericin B; patient 2: 10 mL + 1 g vancomycin + 240 mg gentamicin, 10 mL + 1.5 g meropenem; patient 3: 20 mL + 2 g daptomycin + 2 g meropenem). When a soft tissue defect was present, a muscle flap was performed at the discretion of a plastic surgeon (Table 1). After sampling, patients received empirical antimicrobial therapy intravenously (vancomycin and meropenem). According to microbiological results, antibiotics were adjusted and continued for 3 to 6 months, depending on the joint affected.

**Table 1:** Demographics of all included periprosthetic joint infection (PJI) cases treated with a DAIR procedure. Values are count (%) unless otherwise stated

	Total n = 176	Bead group n = 102	Control group n = 74	Difference in % points or median (CI)
Age, median (IQR)	79 (71–87)	76 (67–83)	83 (76–89)	–5.6 (–9.2 to –2.1)
Female sex	90 (51)	60 (59)	30 (41)	18% (11 to 26)
BMI, median (IQR)	30 (25–34)	31 (26–36)	29 (25–32)	2.5 (0.1 to 4.9)
ASA, median (IQR)	3 (2–3)	3 (2–3)	2 (2–3)	0.1 (–0.1 to 0.3)
Preoperative eGFR < 90	76 (43)	60 (59)	16 (22)	37% (29 to 46)
Joint				
Hip	70 (40)	36 (35)	34 (46)	–11% (–18 to –3)
Knee	106 (60)	66 (65)	40 (54)	11% (3 to 18)
Type of procedure				
DAIR after primary	91 (52)	39 (38)	52 (70)	–32% (–39 to –25)
DAIR after revision	85 (48)	63 (62)	22 (30)	32% (25 to 39)
Previous septic revision(s)	62 (35)	48 (47)	14 (19)	28% (21 to 35)
Number of previous surgeries, median (IQR)	0 (0–3)	2 (0–5)	0 (0–1)	1.2 (–0.2 to 2.5)
JS–BACH classification				
Uncomplicated	79 (45)	34 (33)	45 (61)	–28% (–35 to –20)
Complex	97 (55)	68 (67)	29 (39)	28% (20 to 35)
Type of PJI				
Early postoperative PJI	67 (38)	30 (29)	37 (50)	–22% (–28 to –13)
Acute hematogenous PJI	46 (26)	28 (27)	18 (24)	3% (–4 to 10)
Chronic PJI	63 (36)	44 (43)	19 (26)	18% (10 to 25)
Bacteremia	17 (10)	15 (15)	2 (3)	12% (8 to 16)
Median surgical time, min. (IQR)	94 (71–123)	100 (77–126)	80 (61–106)	21 (7 to 34)
Exchange of mobile parts	135 (77)	86 (84)	49 (66)	18% (12 to 25)
Muscle flap				
Previous muscle flap	10 (6)	8 (8)	2 (3)	5% (2 to 8)
New muscle flap	20 (11)	12 (12)	8 (11)	1 (–4 to 6)
Median follow-up, months (IQR)	53 (28–79)	39 (23–57)	85 (55–112)	–44 (–54 to –34)

### Outcomes

Reinfection was defined as (i) subsequent revision due to infection, and/or (ii) identification of the identical or different microorganism in the affected joint, and/or (iii) the need for suppressive antimicrobial therapy, and/or (iv) a chronic infection without antimicrobial therapy (chronic sinus tract). All subsequent revision surgeries after DAIR during our follow-up period (septic and aseptic revisions) were recorded as reoperations. Heterotopic ossification (HO) was diagnosed on the most recently performed radiograph after DAIR. Wound drainage was defined as prolonged leakage ≥ 4 days postoperatively. A calcium concentration of > 2.6 mmol/L was interpreted as hypercalcemia. If only a partial exchange of mobile parts (e.g., head without liner exchange) was performed, it was considered as no exchange.

### Statistics

Continuous variables were expressed by mean and standard deviation (SD), categorical variables as absolute and relative frequencies (percentage). Univariable logistic regression analyses were performed on the use of CS beads. Kaplan–Meier analyses were used to assess infection-free and revision-free survival in both groups; univariable Cox regression analyses were conducted with censored data from follow-up after recurrence of PJI or when lost to follow-up on the use of CS beads. Estimated parameters are described with 95% confidence intervals (CI). P values < 0.05 were considered significant. For statistical analyses, the software package XLSTAT (Statistical and Data Analysis Solution, New York, USA) was used.

### Ethics, funding, use of AI, and disclosures

This study was approved by the hospital governance department and was exempt from requiring ethical approval as per the Health Research Authority decision tool. It was conducted in accordance with the Declaration of Helsinki. This research did not receive any funding. AI was not used. The authors have no competing interests to declare. Complete disclosure of interest forms according to ICMJE are available on the article page, doi: 10.2340/17453674.2024.42360

## Results

**Figure 3:**
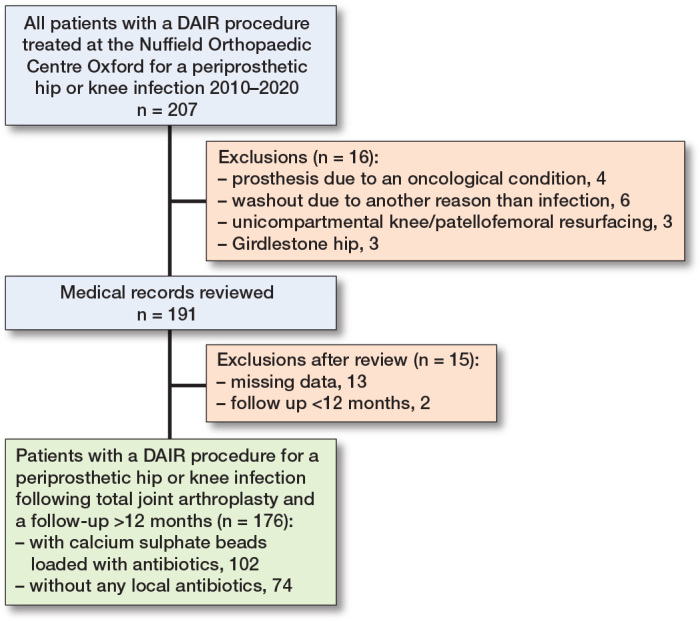
Flowchart illustrating patient selection.

Out of 207 patients undergoing DAIR, 16 were excluded due to exclusion criteria and 15 due to missing data or short follow-up (Figure 3). Thus, 176 patients undergoing DAIR were included: 102 (58%) in the bead group and 74 (42%) in the control group. Demographics and surgical characteristics are listed in Table 1. The most common causative microorganism was *Staphylococcus aureu*s (n = 48; 27%), followed by coagulase negative staphylococci (n=26; 15%), and streptococci (n = 23; 13%, Table 2).

**Table 2:** Distribution of identified microorganisms. Values are count (%)

Microorganisms	n	All PJIs		OR (CI)	Early postoperative PJIs		Acute hematogenous PJIs		Chronic PJIs	
		Bead group (n = 102)	Control group (n = 74)		Bead group (n = 30)	Control group (n = 37)	Bead group (n = 28)	Control group (n = 18)	Bead group (n = 44)	Control group (n = 19)
*S. aureus*	48	30 (29)	18 (24)	1.3 (0.7–2.6)	6	10	13	5	11	3
CoNS	26	15 (15)	11 (15)	1.0 (0.4–2.3)	4	8	2	1	9	2
Streptococcus spp.	23	11 (11)	12 (16)	0.6 (0.3–1.5)	2	2	7	7	2	3
Enterobacteriaceae	11	6 (5.9)	5 (6.8)	0.9 (0.3–3.0)	1	3	2	2	3	0
Enterococcus spp.	3	1 (1.0)	2 (2.7)	0.4 (0.0–4.0)	0	2	0	0	1	0
Cutibacterium spp.	3	0 (0)	3 (4.1)	^a^	0	0	0	0	0	3
*Morganella morganii*	3	3 (2.9)	0 (0)	^a^	0	0	0	0	3	0
*Proteus mirabilis*	2	1 (1.0)	1 (1.4)	0.7 (0.0–11.8)	0	1	0	0	1	0
*Pseudomonas aeruginosa*	2	2 (2.0)	0 (0)	^a^	0	0	0	0	2	0
*Bacteroides fragilis*	1	0 (0)	1 (1.4)	^a^	0	0	0	0	0	1
*Corynebacterium striatum*	1	1 (1.0)	0 (0)	^a^	0	0	0	0	1	0
Polymicrobial	3	26 (25)	17 (23)	1.1 (0.6–2.3)	15	8	1	2	10	7
Including *S. aureus*	19	11 (42)	8 (47)	1.7 (0.6–5.0)	9	3	0	1	2	4
Culture negative	10	6 (5.9)	4 (5.4)	1.1 (0.3–4.0)	2	3	3	1	1	0

OR = odds ratio, *S.aureus = Staphylococcus aureus*; CoNS = coagulase negative staphylococci; PJIs = periprosthetic joint infections.

a No events in one group.

The reinfection rates in early postoperative, acute hematogenous, and chronic PJIs were 25% (17/67), 39% (18/46), and 49% (31/63), respectively. Early postoperative PJIs had significantly lower rates of recurrent infection compared with chronic infections (difference 24%, CI 16–32).

A revision after DAIR was required in 67/176 (38%) patients. The reoperation rates in early postoperative, acute hematogenous, and chronic PJIs were 27% (18/67), 39% (18/46), and 49% (31/63), respectively. Early postoperative PJIs had statistically significantly lower rates compared with chronic PJIs (difference 22%, CI 14-–31).

Of the 3 patients who received additional antibiotics specified by the infectious diseases physicians (rather than the standard vancomycin and meropenem), 1 (patient 3: 20 mL + 2 g daptomycin + 2 g meropenem) had a persistent infection with *Enterococcus faecium* (VRE) and *Enterobacter cloacae* complex. The remaining 2 patients were successfully treated.

### Comparison of groups

**Figure 4:**
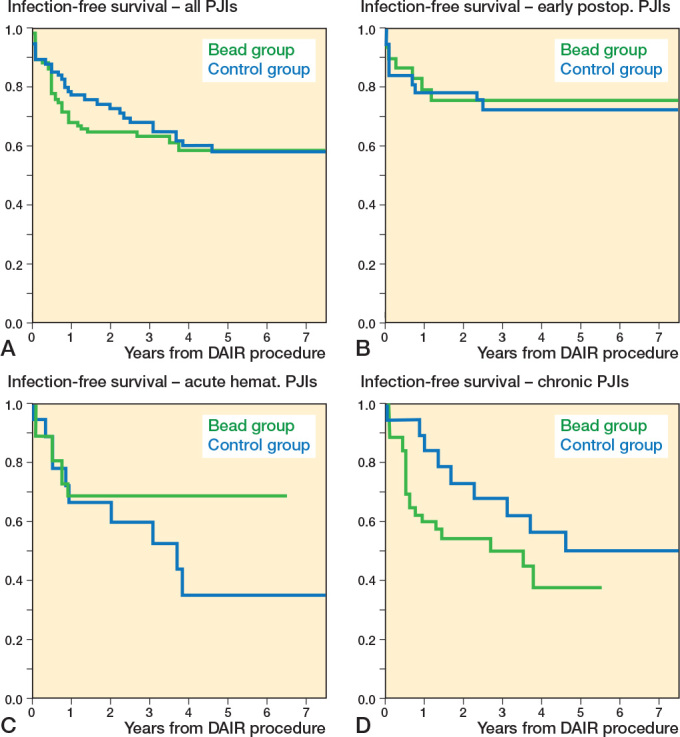
Comparison of Kaplan–Meier infection-free survival curves between the bead (green) and control group (blue) in (A) all included case (HR 1.1, CI 0.7–1.8), (B) early postoperative (HR 0.9, CI 0.3–2.4), (C) acute hematogenous (HR 0.5, CI 0.2–1.4), and (D) chronic periprosthetic joint infections (HR 1.7, CI 0.8–3.7). CI = 95% confidence interval, HR = Hazard ratio, PJI = periprosthetic joint infection.

*Reinfection rate.* 36% (37/102) in the bead group and 39% (29/74) in the control group (OR 0.9, CI 0.5–1.6, Table 3) had reinfections. In the bead group, the infection-free survival probability at 1 year was 68% (CI 59–77), at 2 years 65% (CI 55–73), and at 5 years 59% (CI 48–70). In the control group, it was 77% (CI 67–87) at 1 year, 73% (CI 63–83) at 2 years, and 58% (CI 46–70) at 5 years. No difference was seen between the 2 Kaplan–Meier curves (HR 1.1, CI 0.7–1.8, Figure 4). In the 2 groups, similar reinfection rates were observed in JS-BACH uncomplicated cases (bead: 18% [6/34]; control: 29% [13/45]), complex patients (bead: 46% [31/68]; control: 55% [16/29]), DAIRs after primaries (bead: 23% [9/39]; control: 31% [16/52]), and DAIRs after revisions (bead: 44% [28/63]; control: 59% [13/22]). In acute haematogenous infections, a lower reinfection rate was observed in the bead group (29%) compared with the control group (56%, OR 0.3, CI 0.1-1.1), but not at a statistically significant level. Kaplan–Meier analysis of acute hematogenous PJIs showed a statistically non-significantly higher infection-free survival rate in the bead group (HR 0.5, CI 0.2–1.4).

**Table 3:** Complications of all included PJI cases treated with a DAIR procedure and comparison between different infection types. No case of wear was observed. Values are count (%)

Complications	n	All PJIs			Early postoperative PJIs			Acute hematogenous PJIs			Chronic PJIs		
		Bead group	Control group	OR (CI)	Bead group	Control group	OR (CI)	Bead group	Control group	OR (CI)	Bead group	Control group	OR (CI)
Reinfection	66	37 (36)	29 (39)	0.9 (0.5–1.6)	7	10	0.8 (0.3–2.5)	8	10	0.3 (0.1–1.1)	22	9	1.1 (0.4–3.3)
Reoperation^a^	67	35 (34)	32 (43)	0.7 (0.4–1.3)	8	10	1.0 (0.3–2.9)	7	11	0.2 (0.1–0.8)	20	11	0.6 (0.2–1.8)
Hypercalcemia	9	9 (8.8)	0 (0)	^b^	1	0	^b^	3	0	^b^	5	0	^b^
Wound drainage	34	17 (17)	17 (23)	0.7 (0.3–1.4)	5	12	0.4 (0.1–1.4)	2	2	0.6 (0.1–4.8)	10	3	1.6 (0.4–6.5)
HO	10	8 (7.8)	2 (2.7)	3.1 (0.6–15)	3	0	^b^	0	1	^b^	5	1	2.3 (0.3–21)
Death	35	19 (19)	16 (22)	0.8 (0.4–1.7)	5	11	0.5 (0.1–1.6)	9	1	8.1 (0.9–70)	5	4	0.5 (0.1–2.0)
due to PJI	3	3 (2.9)	0 (0)	^b^	0	0	^b^	3	0	^b^	0	0	^b^

HO = heterotopic ossification; PJIs = periprosthetic joint infection. OR = odds ratio, CI = confidence interval.

a Reoperations due to reinfection (n = 60), aseptic loosening (n = 2), arthrofibrosis (n = 2), dislocation (n = 1), subsidence and HO (n = 1), and hinge fracture (n = 1).

b No events in the control group or overall.

**Figure 5:**
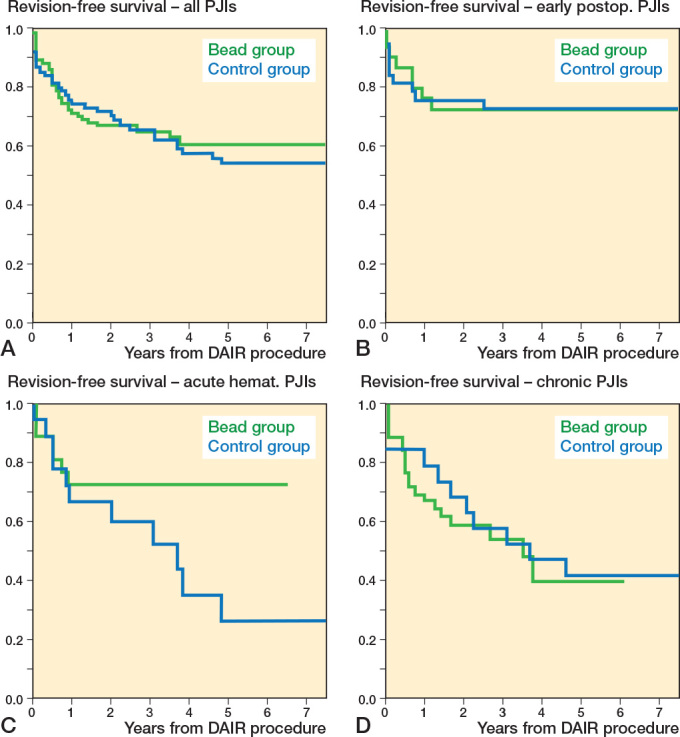
Comparison of Kaplan–Meier revision-free survival curves between the bead (green) and control group (blue) in (A) all included case (HR 1.1, CI 0.7–1.9), (B) early postoperative (HR 1.2, CI 0.5–3.1), (C) acute hematogenous (HR 0.5, CI 0.2–1.3), and (D) chronic periprosthetic joint infections (HR 1.5, CI 0.7–3.4). CI = 95% confidence interval, HR = Hazard ratio, PJI = periprosthetic joint infection.

*Reoperation rate.* In 34% (35/102) in the bead group and 43% (32/74) in the control group, a reoperation was required (OR 0.7, CI 0.4–1.3, Table 3). The revision-free survival probability was 71% (CI 62–80) at 1 year, 67% (CI 57–76) at 2 years, and 60% (CI 49–71) at 5 years in the bead group, and 74% (CI 64–84), 70% (CI 60–81), and 54% (CI 42–66) in the control group. Kaplan–Meier curves showed no statistically significant difference between the groups (HR 1.1, CI 0.7–1.9, Figure 5). In acute hematogenous PJIs, fewer reoperations were observed in the bead group (25% [7/28]) compared with the control group (61% [11/18]; OR 0.2, CI 0.1–0.8). Kaplan–Meier analysis showed a statistically non-significantly higher revision free survival rate in the bead group (HR 0.5, CI 0.2–1.3).

*Transient asymptomatic hypercalcemia.* Transient asymptomatic hypercalcemia was recorded in 8 patients of the bead group (CS beads of 10 mL [n = 4], 20 mL [n = 3], and 40 mL [n = 1] antibiotic-loaded rapid cure powder; Table 3). In addition, 1 female 60-year-old patient with a chronic PJI caused by *Staphylococcus aureus* and a background of primary hyperparathyroidism and chronic kidney disease (stage 3a), who was treated with a DAIR procedure, CS beads of 20 mL antibiotic-loaded rapid cure powder, and a medial gastrocnemius muscle flap developed symptomatic hypercalcemia and painful necrotic skin lesions, for which the working diagnosis was calciphylaxis secondary to hyperparathyroidism (the differential diagnosis was heparin-induced skin necrosis). These progressed over 4 months and despite medication adjustment and normocalcemia, she died 4 months postoperatively.

**Figure 6:**
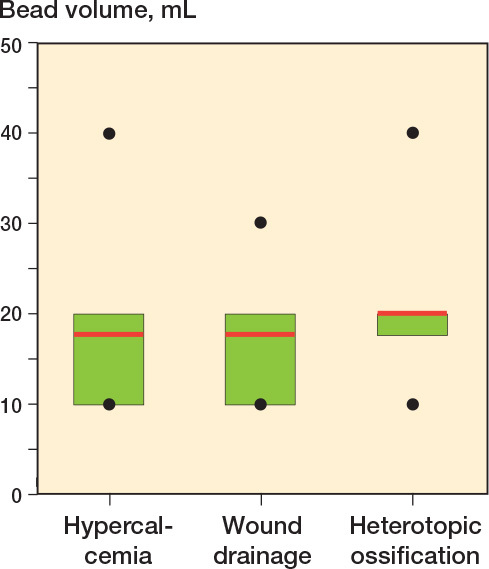
Boxplots of bead volume in patients with hypercalcemia (n = 9), wound drainage (n = 17), and heterotopic ossification (n = 8) in the bead group. The red line represents the mean bead volume, the green box the interquartile range, the black dots the minimum and maximum. Median bead volume was 20 mL for all complications.

*Wound drainage.* No difference was seen between the 2 groups (bead group: 17% [17/102]; control group: 23% [17/74], OR 0.7, CI 0.3–1.4, Table 3). When CS beads of 10 mL, 20 mL, 30 mL, and 40 mL antibiotic-loaded rapid cure powder were applied, the drainage rate in the bead group was 15% (8/53), 19% (8/43), 25% (1/4), and 0% (0/2), respectively (Figure 6).

*HO.* 8 patients in the bead group (8%, 10 mL [n = 2], 20 mL [n = 5], 30mL [n = 1]) and 2 patients in the control group had HO (3%, OR 3.1, CI 0.6–14.9, Table 3). 1 of these patients (13%) was revised due to subsidence and HO (20 mL). No revision due to wear was performed in our study period.

## Discussion

This is the largest series with the longest follow-up to date evaluating the effectiveness of CS beads in patients undergoing DAIR. We aimed to compare the effect of CS beads loaded with antibiotics on infection eradication in DAIR relative to DAIR without local antibiotics delivery.

We found that the reinfection and reoperation rates after a DAIR procedure were similar between the bead and the control group. However, sub-analysis showed an improved outcome when CS beads were used in acute hematogenous PJIs. Although the failure rate was high in our control group (56%), similar results were reported in patients with this type of infection in the literature (51–60%) [22-24]. Hence, it appears that CS beads may improve DAIR outcome in acute hematogenous PJIs. This may be explained by the eradication of new planktonic microorganisms originating from the still existing infection source (endocarditis, pneumonia, etc.) in these infections (where continuous seeding from persistent bacteremia is possible) [24] and, therefore, a new hematogenous spread to the affected joint may be avoided. This would also explain the similar reinfection rate in acute hematogenous infections treated with CS beads and early postoperative PJIs (29% vs 27%). Another possible explanation could be the higher mortality rate in the bead group (32% vs 6%). Patients may have died before reinfection occurred. However, 4 of the 9 deaths already had a documented reinfection before death. 3 patients died within 1 month following DAIR due to sepsis, and 2 patients after a long time period following DAIR (22 months and 59 months; not related to infection). Hence, we do not believe that the higher mortality rate had a major impact on our results. Nevertheless, the exact reason for the improved DAIR outcome in acute hematogenous PJIs remains unclear.

In general, degradable CS beads loaded with antibiotics are used with the aim of improving DAIR outcome. However, their use when performing DAIR remains controversial (Table 4) [8-15]. Reinisch et al. demonstrated a lower failure rate in patients treated with DAIR and CS beads (15% [4/27]; without beads: 79% [11/14], P < 0.0001) in their retrospective cohort study of 41 hip PJIs [13]. In another consecutive series of 62 early postoperative PJIs undergoing DAIR including CS beads application, a low reinfection rate of 23% (n = 14/62) was observed [9]. Kallala et al. demonstrated a reinfection rate of only 9% (6/68) following debridement with CS beads in their large series of 755 cases undergoing revision lower limb arthroplasty [11]. However, their indication for a DAIR procedure was not only PJI. Gramlich et al. again described a lower reinfection rate when CS beads were applied during DAIR (bead: 35% [8/23] vs control: 82% [27/33], P < 0.001), but it is noteworthy that only patients with chronic knee PJIs unsuitable for multistage procedures were included in their study [15]. They concluded that DAIR with CS beads is a reasonable treatment option in this special population.

**Table 4:** Comparison of the literature regarding outcome of DAIR procedures with CS beads loaded with antibiotics. All listed studies are retrospectively designed, except the study by Kallala et al. [11]

Literature	DAIRs/PJIs (n)	Infection type^a^	Joint	Mean FU (range)^a^	Beads^a^	Local antibiotics^a^	Hypercalcemia (%)	Wound drainage (%)	HO (%)	Reinfection (%)
Flierl [8]	33	14 EP 19 AH	27 TKA 6 THA	12.7 m (3–30)	Stimulan Rapid Cure^c^	10 mL mixed with 1 g vancomycin, 1.2 g tobramycin	NA	NA	NA	48
Kallala [11]	68	NA^b^	30 TKA 19 THA	35 m (0–78)	Stimulan^d^	10 mL mixed with 1 g vancomycin, 240 mg tobramycin	3	6	NA	9
Gramlich [10]	42	Chronic	12 TKA 19 THA 11 knee arthrodesis	23 m (SD 10.3)	Osteoset ^e,^ Osteoset T^e,^ or Herafill G40^f^	mixed with vancomycin, ceftriaxone, or colistin; mixed with 4% tobra-mycin sulfate; mixed with 1% genta-micin sulfate	NA	NA	NA	26
Gramlich [15]	23	Chronic	TKA	NA	Osteoset ^e,^ Osteoset T^e,^ or Herafill G40^f^	mixed with vancomycin, ceftriaxone, or colistin; mixed with 4% tobra-mycin sulfate; mixed with 1% genta-micin sulfate	NA	NA	NA	35
Tarity [12]	20	EP, AH	12 TKA 8 THA	24 m (NA)	NA	10 mL mixed with 1 g vancomycin, 1.2 g tobramycin	NA	NA	NA	45
Reinisch [13]	27	EP, AH	THA	Min 12 m	Osteoset and Osteoset T ^e^	3x25 mL packs loaded with 2 g vancomycin each (Osteoset) or 2 g ceftriaxone	NA	NA	NA	15
Piovan [14]	17	12 EP 5 AH	TKA	16 m (12–37)	Stimulan^d^	10 mL mixed with 1 g vancomycin and 240 mg gentamicin or 10 mL mixed with 1 g vancomycin and 240 mg tobramycin	0	0	0	12
Indelli [9]	62	acute PJIs	37 TKA 19 THA 6 TSA	(24–84)	Stimulan^d^	According to the anti-biogram or molecular testing results	0	6	2	23
Present study	102	67 EP 46 AH 63 chronic	106 TKA 70 THA	61 m (12–147)	Stimulan Rapid Cure^d^	10 mL mixed with 1 g vancomycin and 240 mg gentamicin	9	17	8	36

DAIR = debridement, antibiotics, and implant retention; FU = follow-up; HO = heterotopic ossification; EP = early postoperative PJI; AH = acute hematogenous PJI; TKA = total knee arthroplasty; THA = total hip arthroplasty; TSA = total shoulder arthroplasty; NA = not available; m = months, min = minimum.

a As described in the studies.

b DAIR indication: not only PJI.

c Biocomposites Inc, Wilmington, NC, USA

d Biocomposites, Keele, UK

e Wright Medical Technology Inc, Arlington, TN, USA

f Heraeus Medical GmbH, Wehrheim, Germany

Other study groups were not able to demonstrate such good results. Flierl et al. showed a high reinfection rate (48% [16/33]) when CS beads were added during DAIR [8]. In another retrospectively conducted, small, matched cohort study of 20 DAIR procedures, the application of CS beads did not reduce the reinfection rate (bead: 45% [9/20] vs control: 25% [5/20]; P = 0.2) [12]. However, a comparison between these studies and our cohort is difficult. DAIR indications (type of infections, infection definitions, etc.), host factors, definitions of DAIR success/failure, length of follow-up, microorganism spectrums, and antimicrobial therapy varied tremendously among them. Furthermore, most had an inadequate sample size.

Complications after CS beads application are uncommon. Hypercalcemia is a potential side effect, especially when higher volumes (> 40 mL powder) are used [11]. Although the majority of patients in our cohort and in the literature were asymptomatic, some may require active treatment [11]. Untreated hypercalcemia can lead to symptoms such as convulsions, coma, and cardiac arrest. Therefore, calcium levels and renal function should be monitored pre- and postoperatively. In our cohort, some of the patients who only received CS beads of 10 mL powder developed hypercalcemia, demonstrating the common high level of comorbidity of patients with PJI. In patients with renal insufficiency, parathyroid disease, and critical illness, we now carefully consider the risks and benefits of using CS beads [17].

Wound drainage is an often-reported issue when using CS beads, but the majority of our patients received a bead volume made of ≤ 20 mL powder, which does not increase the overall risk of wound drainage.

### Limitations

This study is limited by its retrospective nature and comparison of the CS bead group (2015–2020) with a historical cohort (2010–2014). However, surgical techniques of DAIR, diagnostic methods, and empirical and definitive antimicrobial therapy did not change from 2010 to 2020 at our institution except for the introduction of CS beads use in 2015. Furthermore, demographics between the 2 groups are heterogeneous, demonstrating the changing nature of PJI with patients now having increased BMIs and more comorbidities. Additionally, patients in the bead group were more complex, had more previous revisions, and more often a chronic infection. Nevertheless, other comparative studies of this type described substantial variabilities in their microorganism spectrum [13,14], which was very similar between the 2 groups in our cohort. Finally, the sample size in our sub-analyses was small. Hence, further investigation with a larger, multi-center trial is recommended.

### Conclusion

Although DAIR and CS beads did not significantly improve outcome in early postoperative and chronic infections, acute hematogenous PJIs showed higher success rates when CS beads loaded with antibiotics were used with similar results compared with early postoperative infections. Complications are rare and to reduce these we suggest calcium levels and renal function should be monitored pre- and postoperatively.

## Notes

IKS: conceptualization, methodology, investigation, data curation, formal analysis, validation, visualization, writing – original draft, writing – review & editing. AP: conceptualization, investigation, writing – review & editing. AH: data curation, investigation, writing – review & editing. MM: conceptualization, investigation, supervision, writing – review & editing. AA, AT, BY: conceptualization, investigation, writing – review & editing. BK: conceptualization, methodology, supervision, formal analysis, validation, writing – review & editing.

Handling co-editors: Bart A Swierstra and Robin Christensen

*Acta* thanks Daniël Janssen and Christian Thomas Pollmann for help with peer review of this manuscript.
